# SGCNCMI: A New Model Combining Multi-Modal Information to Predict circRNA-Related miRNAs, Diseases and Genes

**DOI:** 10.3390/biology11091350

**Published:** 2022-09-13

**Authors:** Chang-Qing Yu, Xin-Fei Wang, Li-Ping Li, Zhu-Hong You, Wen-Zhun Huang, Yue-Chao Li, Zhong-Hao Ren, Yong-Jian Guan

**Affiliations:** 1 School of Information Engineering, Xijing University, Xi’an 710123, China; 2 College of Grassland and Environment Sciences, Xinjiang Agricultural University, Urumqi 830052, China; 3 School of Computer Science, Northwestern Polytechnical University, Xi’an 710129, China

**Keywords:** circRNA–miRNA interaction, circRNA–cancer, graph convolution network, miRNA, k-mer

## Abstract

**Simple Summary:**

With the development of circRNA–miRNA-mediated models, circRNAs have been shown to play a prominent role in the development and treatment of diseases such as cancer, and unearthing potential miRNA-associated circRNAs may provide new insights and ideas for the diagnosis and treatment of complex diseases such as cancer. Large-scale prediction using computer technology can provide an a priori guide to biological experiments and save costs. This paper presents the third computational method in this field with the highest accuracy to date, and we also collected and integrated high-quality datasets from the current database, which we believe will allow future computational innovations to develop.

**Abstract:**

Computational prediction of miRNAs, diseases, and genes associated with circRNAs has important implications for circRNA research, as well as provides a reference for wet experiments to save costs and time. In this study, SGCNCMI, a computational model combining multimodal information and graph convolutional neural networks, combines node similarity to form node information and then predicts associated nodes using GCN with a distributive contribution mechanism. The model can be used not only to predict the molecular level of circRNA–miRNA interactions but also to predict circRNA–cancer and circRNA–gene associations. The AUCs of circRNA—miRNA, circRNA–disease, and circRNA–gene associations in the five-fold cross-validation experiment of SGCNCMI is 89.42%, 84.18%, and 82.44%, respectively. SGCNCMI is one of the few models in this field and achieved the best results. In addition, in our case study, six of the top ten relationship pairs with the highest prediction scores were verified in PubMed.

## 1. Introduction

Circular RNA (circRNA) is a special kind of single-stranded circular endogenous non-coding RNA (ncRNA). Recent research shows that endogenous circRNAs are widely distributed in mammalian cells and involved in transcriptional and posttranscriptional gene expression regulation [1]. CircRNA was first discovered in RNA viruses as early as 1976 [2], and in 1979, Hsu et al. provided electron microscopic evidence for the circular form of RNA [3]. Over the following three decades, only a handful of circRNAs were discovered by chance [4,5,6], and due to their low levels of expression, circRNAs were typically considered to be products of “noise” of an abnormal RNA splicing process, which resulted in circRNAs not receiving corresponding attention.

However, since 2010, with the development of RNA-seq technologies and specialized computational pipelines, many circRNAs have been widely recognized and discovered in eukaryotes, such as mice [7], archaea [8], and humans [9]. With the progress in circRNA research, many circRNAs have been proven to present tissue-specific expression patterns and have specific biological functions [10]. Emerging experimental results show that endogenous circRNAs widely exist in mammals and can work as miRNA sponges, which means that circRNAs reverse the inhibitory effect of the miRNA on its target gene and consequently repress their function [11]. At present, many types of research have indicated the association between miRNA sponges (circRNAs) and human diseases.

CircRNAs have a prominent role in cancer diagnosis and treatment [12]. For example, in bladder cancer studies, circ-ITCH acted as a miRNA sponge to inhibit bladder cancer progression by directly regulating p21 and PTEN in combination with miR-17 and miR-224. Circ-ITCH expression was also lower than normal in bladder cancer tissues [13]. In addition, high expression of another circRNA, circ-TFRC, was detected in bladder cancer patients, which means that circ-TFRC promotes bladder cancer progression by binding to miR-107 [14]. CircCCDC9 expression was significantly lower than normal in gastric cancer tissue samples, and the study confirmed that circCCDC9 inhibits the progression of gastric cancer by regulating CAV1 in combination with miR-6729-3p [15]. CircRNA also plays an important role in the development of renal cell carcinoma (RCC) by regulating CDKN3/E2F1 in combination with miR-127-3p [16]. These results suggest that investigating circRNA–miRNA interactions could be key to diagnosing and addressing complex diseases such as cancer.

Compared with traditional biological experimental methods, which are limited to small scales and require lots of labor and time, using computational models to predict the association between molecules can provide the basis for biological experiments at a low cost. At present, many computational methods have been proposed and applied to predict the correlation between different molecules. For example, Wang et al. proposed a model, SAEMDA, through a new unsupervised training method named Stacked Autoencoder to predict miRNA–disease associations [17]. Ren et al. developed a model named BioChemDDI, which combines a Natural Language Processing algorithm and Hierarchical Representation Learning to effectively extract information, employed Similarity Network Fusion to fuse multiple features, and finally applied a deep neural network to obtain the predicted results [18]. Wang et al. proposed a new method that extracts deep features of molecular similarity through a deep convolutional neural network and sends them to an extreme learning machine classifier to identify potential circRNA–miRNA associations [19]. Such computational methods have achieved gratifying results and provided an experimental basis for further wet experiments.

Compared with related fields, where new circRNA molecules are constantly discovered and the nomenclature is not fully standardized, there are a few computational methods that predict associations between circRNAs and miRNAs. However, with the rapid development of high-throughput sequencing technology, a large number of databases have been developed to store circRNA-related information, such as circR2Disease [20], circRNAdisease [21], circbank [22], and circBase [23]. The circR2Disease database is a high-quality database containing detailed information about circRNA. The latest version contains about 750 circRNA–disease associations between more than 600 circRNAs and 100 diseases. The circRNAdisease database manually collects verified circRNA–disease pairs from the PubMed database by retrieving circRNA and disease keywords. Circbank is a comprehensive database that contains multiple characteristics of circRNA; more than 140,000 circRNAs from different sources can be retrieved by users from the circbank database. The circBase database is one of the early databases to collect circRNA information, including circRNA data, evidence to support circRNA expression, and scripts for identifying known and new circRNAs in sequencing data. The establishment of these databases has provided the materials for predicting associations between circRNAs and miRNAs by using computational methods.

At present, only few models have been proposed, and the predicted results were confirmed in PubMed. Compared with other fields, there are few computational prediction models for circRNA–miRNA interaction prediction. Therefore, it is urgent to develop new and effective prediction methods for circRNA–miRNA association prediction.

According to our understanding, there are some obstacles to using computational methods to predict circRNA–miRNA interactions: (i) The length of circRNA and miRNA sequences varies greatly, resulting in redundancy or sparsity of biological information collected. (ii) A network composed of confirmed circRNA–miRNA associations is difficult to connect, which means it is difficult to extract effective features from relatively isolated nodes. (iii) The data on circRNA–miRNA interactions are scattered among different databases, so it is difficult to collect comprehensive and reliable data. To solve these problems, we developed a model, SGCNCMI, to predict circRNA–miRNA interactions based on multi-source feature extraction and graph representation learning with a layer contribution mechanism. Specifically, we first adopt a K-mer algorithm to extract the internal attribute features in the sequence by taking the most appropriate K value for different RNAs, and to make full use of RNA molecular biological information, two kinds of kernel functions are added to enrich semantic descriptors. Secondly, we introduce the Sparse Autoencoder (SAE) with a sparsity penalty term to process semantic descriptors to obtain the most valuable molecular biological attribute information. Next, we apply a multilayer graph convolutional neural network (GCN) to project the circRNA–miRNA interaction network into a new space to capture non-linear interactions and hidden associations. Meanwhile, we include a layer contribution mechanism in the graph convolutional layer to ensure the maximum contribution of GCN in each layer. Finally, the predicted score of each pair of circRNA–miRNA is obtained from the inner product of the corresponding potential vectors.

Notably, our model supports training and prediction using two types of training data, one based on circRNA–miRNA molecular sequences and known association data and the other based on circRNA as a cancer marker. This means that our model can be trained and predicted from the perspective of both potential molecular relationships and data on associations between clinical disease and markers.

As a result, in a five-fold cross-validation experiment to measure the ability of the model, 89.42% AUC and 88.87% AUPR were obtained by SGCNCMI, and in the circRNA–miRNA interaction dataset test, the performance of SGCNCMI exceeded that of the only other model at present. In addition, 84.18% AUC and 84.83% AUPR were obtained by SGCNCMI in the circRNA–cancer dataset test, and 82.44% AUC and 85.55% AUPR were obtained in the circRNA–gene dataset. Meanwhile, 7 of the 10 pairs with the top predicted scores of the circRNA–miRNA interaction dataset test was verified in PubMed. Obviously, our model, SGCNCMI, is one of the few accurate and reliable prediction models in the field of circRNA–miRNA interaction prediction and is expected to become a powerful candidate model for biological experiments.

## 2. Materials and Methods

### 2.1. Dataset

As research progresses, a number of positive circRNA–miRNA correlations have been identified, and various databases have been established. The CircR2Cancer database [24] is an online database that gathers experimentally validated circRNA–cancer and circRNA–miRNA associations reported in published papers. After rigorous screening, we obtained 318 circRNA–miRNA relational pairs between 238 circRNAs and 230 miRNAs.

At present, the techniques for predicting target gene binding sites are well developed, allowing the selection of candidates that closely match the binding sites, with high accuracy for most binding sites, and the vast majority of these predictions were eventually validated in subsequent experiments. Predicting target gene binding sites is already widely used in a variety of methods and tools; for example, CircInteractome [25] uses a well-established TargetScan Perl script to analyze miRNAs that may be associated with circRNA. These data are extremely valuable. The circBank database [22] performs binding site predictions for 140,790 human circRNAs and 1917 miRNAs using Miranda [26] and TargetScan [27] techniques, resulting in 42,917 relationships with more than five binding sites and 3545 relationships with more than one binding site. We selected the top 9589 pairs of circRNA–miRNA relationship pairs with the highest scores. These data were used in the first computational method [28] in the field and partially validated in PubMed.

After combining data from both databases, we eventually obtained 9905 pairs of high-quality relationships for training in our methods, and for ease of description, we identified this dataset as CMI-9905.

To test SGCNCMI’s ability to predict the association between markers and underlying diseases, we downloaded 1049 experimentally supported circRNA–cancer relationship pairs from the Lnc2Cancer database [29] of 743 circRNAs and 70 cancers.

In addition, we downloaded circRNA–gene-associated data from the TransCirc [30] database and selected the top 2000 pairs with the highest confidence scores as training data.

### 2.2. CircRNA and miRNA Sequence Similarity Based on K-mer

Counting RNA sequences’ *K-mers* (substrings of length k) is not only an important and common step in bioinformatics analysis but also widely used in computational methods [31,32]. Related studies have indicated that RNA sequences contain abundant biological information. Converting sequence information into a digital vector is an important method to obtain molecular biological information in order to fully explore hidden features in RNA sequences. The *K-mer* sparse matrix is used to represent RNAs’ attribute features in our model.

For a circRNA sequence, we apply the best 5*-mers* as the window to scan the sequence, moving one nucleotide at a time. Due to there being four different nucleotides in circRNA, the window of 5*-mers* will produce 4^5^ vector representations for each circRNA molecule. Therefore, the *K-mer* matrix of circRNA can be represented as follows:

(1) $$KM_{\mathrm{circ}RNA}=2346\times 4^{5}$$

For a miRNA sequence, with an average length of 21 nucleotides, the scan window we use is 2*-mers* to obtain the best vector representations, and the *K-mer* matrix of miRNA is defined as:

(2) $$KM_{miRNA}=962\times 4^{2}$$

**Figure 1:**
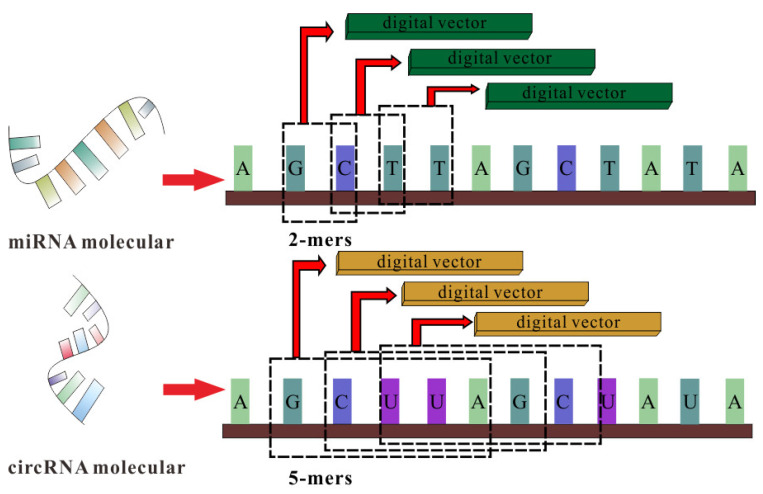
The *K-mer* algorithm for sequence feature extraction.

The details of the *K-mer* algorithm are shown in Figure 1.

### 2.3. Similarity for CircRNA and miRNA

RNAs that can bind to the same molecule often have the same binding sites, which means that a potential unknown association can be inferred by analyzing RNA molecules with the same function. In order to fully express the biological characteristics of RNA molecules, we introduce two kinds of similarities (RNA Gaussian interaction profile kernel similarity and RNA sigmoid kernel similarity) as RNA semantic descriptors.

Firstly, we construct a bipartite graph *B_C__×M_* to represent the 9905 associations between circRNA and miRNA interaction pairs for 2346 circRNAs and 962 miRNAs. In the matrix *B_C__×M_*, *C* and *M* represent the number of circRNAs and miRNAs. When circRNA *i* is related to miRNA *j*, the value of *B_i×j_* is equal to 1 and otherwise equal to 0. Each row and column represent circRNA and miRNA interaction profiles, respectively; the interaction profile binary vector *LP*(*C_i_*) of circRNA *C_i_* is the row corresponding to the circRNA in the adjacent matrix *B_C__×M_*, and the GIP kernel of each circRNA can be calculated as:

(3) $$G_{circRNA}(C_{i},C_{j})=\exp(-\alpha_{c}||LP(C_{i})-LP(C_{j})|{|}^{2})$$

where *C_i_* and *C_j_* denote circRNA *i* and circRNA *j*, G_circRNA_ (*C_i_*, *C_j_*) is the GIP kernel similarity between circRNA *i* and circRNA *j*, and *α_c_* is a variable parameter that controls the bandwidth of the GIP kernel, which is defined as follows:

(4) $$\alpha_{c}=\alpha_{c}´/(\frac{1}{nc}\sum_{i=1}^{nc}||LP(C_{i})|{|}^{2})$$

In this experiment, α_c_’ is defined as equal to 0.5.

Similarly, the GIP kernel similarity between miRNA *m_i_* and miRNA *m_j_* is calculated as

(5) $$G_{miRNA}(M_{i},M_{j})=\exp(-\alpha_{m}||LP(M_{i})-LP(M_{j})|{|}^{2})$$

(6) $$\alpha_{m}=\alpha_{m}´/(\frac{1}{nm}\sum_{i=1}^{nm}||LP(M_{i})|{|}^{2})$$

The sigmoid kernel of each circRNA is defined as follows:

(7) $$K_{circRNA}(C_{i},C_{j})=\tanh\{\beta [\rho (C_{i})]\times \kappa [\rho (C_{j})]\}$$

where *β = 1/V*, and *V* is the dimension of original input data.

In the same way, the sigmoid kernel of each miRNA is defined by the formula below:

(8) $$K_{miRNA}(M_{i},M_{j})=\tanh\{\beta [\rho (M_{i})]\times \kappa [\rho (M_{j})]\}$$

### 2.4. Integrating Attributes and Similarity for circRNA and miRNA

Feature fusion can incorporate more meaningful information from different aspects, which can comprehensively reflect the characteristics of the circRNA and miRNA. In this section, we construct the characteristic fusion matrices of circRNA and miRNA. First, the different types of circRNA similarity (GIPKS and sigmoid kernel) matrixes are combined into one matrix called *F_C_(c_i_,c_j_)* by the following formula:

(9) $$F_{C}(C_{i},C_{j})=\left\{\begin{array}{cc} \frac{G_{circRNA}(c_{i},c_{j})+K_{circRNA}(c_{i},c_{j})}{2} & G_{circRNA}(c_{i},c_{j}),K_{circRNA}(c_{i},c_{j})\neq 0\\ G_{circRNA}(c_{i},c_{j})+K_{circRNA}(c_{i},c_{j}) & otherwise \end{array}\right.$$

In the same way, the miRNA similarity matrix is defined as

(10) $$F_{M}(M_{i},M_{j})=\left\{\begin{array}{cc} \frac{G_{miRNA}(m_{i},m_{j})+K_{miRNA}(m_{i},m_{j})}{2} & G_{miRNA}(m_{i},m_{j}),K_{miRNA}(m_{i},m_{j})\neq 0\\ G_{miRNA}(m_{i},m_{j})+K_{miRNA}(m_{i},m_{j}) & otherwise \end{array}\right.$$

We integrate the attribute feature matrix and similarity feature matrix to obtain the heterogeneous network *H_C__×M_* as follows:

(11) $$H_{C\times M}=\left[\begin{array}{cc} KM_{circRNA} & F_{C}\\ KM_{miRNA} & F_{M} \end{array}\right]$$

### 2.5. Node Feature Extraction Based on Sparse Autoencoder (SAE)

The features extracted from sequence and similarity often have information redundancy or “noise”. In this section, the Sparse Autoencoder (SAE) [33] is used to reconstruct the eigenmatrix. As an unsupervised autoencoder, SAE can effectively learn the hidden features of input vectors, while the introduction of a sparsity penalty term can also learn relatively sparse features well.

SAE is an unsupervised encoder including an input layer hidden layer and output layer. The input layer maps the input data *X* to the hidden layer *L_h_* for encoding, where layer *L_h_* is defined as follows:

(12) $$L_{h}=\sigma (W_{L_{i}}X(i)+b_{L_{i}})$$

where *X(i)* is the original input data, WL is a connection parameter between the input and hidden layers, and *b_Li_* represents an offset of function.

SAE defines *σ()* as the activate function, which can be represented as:

(13) $$\sigma (X)=\frac{1}{\left(1+e^{-X}\right)}$$

The average activation of the activated hidden units can be calculated as:

(14) $${\hat{\rho }}_{h}=\frac{1}{n}\sum_{i=1}^{n}\left[a_{h}(X(i))\right]$$

where *α_h_()* denotes the activation amount of the hidden units.

The sparsity penalty term *P_s_* is added to the target function to keep the hidden layer at low average activation values, which are shown as:

(15) $$P_{s}=\sum_{i=1}^{L_{n}}KL(\rho ||{\hat{\rho }}_{h})$$

where *P_s_* is the sum of the degrees of penalization, ${\hat{\rho }}_{h}$ deviates from *ρ*, and *L_n_* represents the number of units in the hidden layer. KL divergence (Kullback–Leibler) represents the sparsity penalty term of SAE and is defined as follows:

(16) $$KL(\rho ||{\hat{\rho }}_{h})=\rho \log\frac{\rho }{\hat{\rho }}+(1-\rho )\log\frac{1-\rho }{1-{\hat{\rho }}_{h}}$$

where *ρ* is the sparsity parameter of *KL*, which is close to 0; when ${\hat{\rho }}_{h}$ is closer to *ρ*, the value of *KL* is smaller, and when ${\hat{\rho }}_{h}$ is equal to *ρ*, KL is equal to 0; otherwise, it increases monotonically.

With the sparsity penalty term added, the cost function is defined as:

(17) $$F_{\cos t}(W,B)=C_{L}(w,b)+\delta \sum_{i=1}^{L_{n}}KL(\rho ||\hat{\rho })$$

where *δ* is the weight of the sparsity penalty term, and *C_L_(w, b)* is the cost function of each layer, which is calculated by the backpropagation algorithm:

(18) $$w(L)=w(L)-\vartheta \frac{\partial }{\partial w(L)}C_{\cos t}(W,B)$$

(19) $$b(L)=b(L)-\vartheta \frac{\partial }{\partial b(L)}C_{\cos t}(W,B)$$

where *ϑ* denotes the learning rate of the neural networks.

In this work, the heterogeneous network *H* is processed by SAE as the input data, and the final characteristic matrix *D_C__×M_* is generated, where each row of *D_C__×M_* represents the attribute characteristics of the corresponding node.

### 2.6. SGCNCMI

According to the effective application of graph neural networks in the prediction field, we propose a novel prediction model (SGCNCMI) based on a graph convolutional neural network. SGCNCMI can be described in the following six steps: (1) construct a circRNA–miRNA adjacency matrix, (2) use the RNA sequence and functional similarity to generate the node attribute feature representation, (3) use the Sparse Autoencoder (SAE) to further extract features and generate the final node feature representation, (4) apply GCN to map the relationship network diagram to a new space so as to aggregate the features of potentially associated nodes, (5) apply the weighted cross-entropy loss function to train the whole model in an end-to-end manner, and (6) apply an inner product decoder to score each pair of relationships. Next, the implementation details for each step are shown.

In step 1, we integrated known circRNA–miRNA interactions into an adjacency matrix, which contained 9905 processed high-quality interaction pairs between 2346 circRNAs and 962 miRNAs. We treated all of these 9905 interaction pairs as positive edges between circRNA nodes and miRNA nodes, and we also randomly constructed 9905 negative samples to balance the training set to better train the model. Then, all of the positive edges were labeled 1, and all of the negative samples were labeled 0.

In step 2, in order to fully express the attributes of nodes, we tried to combine multi-source information to extract node features and convert the features into digital vectors. First, related studies have confirmed that RNA molecular sequences contain abundant biological attribute information, and we applied the *K-mer* algorithm to process sequences to obtain the underlying feature representation. Due to the difference in the length of RNA sequences, we used *5-mers* for circRNA and *2-mers* for miRNA, and finally, we obtained a 128-dimension circRNA sequence vector and a 16-dimension miRNA sequence vector. Next, based on the assumption that circRNAs with similar functions are likely to be related to miRNAs with similar phenotypes, we increased two kinds of similarity (RNA Gaussian interaction profile kernel similarity and RNA sigmoid kernel similarity) to construct the comprehensive similarity matrix.

In step 3, we used SAE to further process the preliminary multidimensional features. SAE is an unsupervised autoencoder with sparsity penalty terms that can effectively extract potential features from a matrix with redundant information, while the introduction of the sparsity penalty term can obtain more valuable information from the sparse matrix. Finally, we obtained the comprehensive characteristics vectors of each node as below:

(20) $$V_{c}={\left(c_{1},c_{2},\ldots ,c_{2346}\right)}^{⊤}$$

(21) $$V_{m}={\left(m_{1},m_{2},\ldots ,m_{962}\right)}^{⊤}$$

where *c_i_* represents the features of circRNA *i*, and *m_j_* represents the features of miRNA *j*.

In step 4, we transformed the prediction of circRNA–miRNA association into a link prediction problem on a heterogeneous bipartite graph, and GCN was used to effectively learn latent graph structure information and the representations of node attributes from an end-to-end model structure. First, for an undirected heterogeneous bipartite graph *A*, self-connections were added to ensure nodes’ characteristic contributions:

(22) $$\hat{A}=A+I$$

where *A* is the bipartite graph, and I is the identity matrix. In order to promote the contribution of the association relation in the propagation process of the graph convolutional network, we normalized matrix $\tilde{A}$ as follows:

(23) $$\tilde{A}={\tilde{D}}_{}^{-\frac{1}{2}}\hat{A}{\tilde{D}}_{}^{-\frac{1}{2}}$$

where $\tilde{D}$ is calculated as:

(24) $${\tilde{D}}_{ii}=diag(\sum_{}{}_{j}{\tilde{A}}_{ij})$$

**Figure 2:**
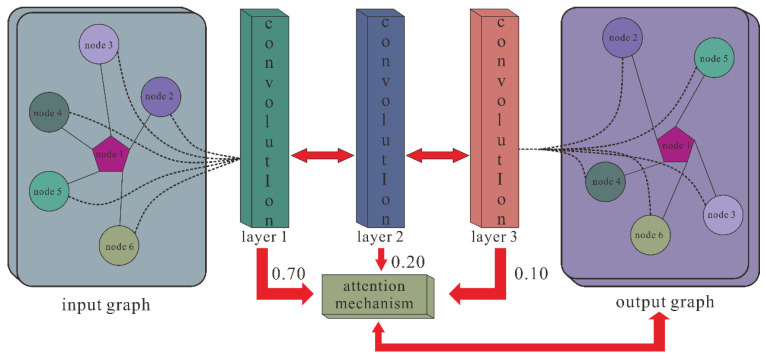
GCN extracted features.

Then, we utilized GCN containing three layers of graph convolutional networks to aggregate node features and generate a corresponding lower-dimensional feature matrix. The specific process is shown in the following formulas:

(25) $$H^{\left(l+1\right)}=\sigma (\tilde{A}H^{\left(l\right)}W^{\left(l\right)})$$

where *H^(l)^* represents the node feature vector of the lth layer, and *H^(0)^* is the comprehensive characteristics vector of each node that is extracted by SAE. *W^(l)^* is the lth layer trainable weight matrix, and *σ()* denotes the ReLU activation function. Meanwhile, to solve the problem that the contributions of different layers’ embeddings are unequal, we introduce the attention mechanism, which is defined as follows:

(26) $$M_{cm}=\sum {}_{l}n_{l}H^{\left(l\right)}$$

where *n_l_* is the weight parameter, which is auto-learned by the graph convolutional network, and *M_cm_* is the final embedding representation obtained by GCN. The GCN extraction process is shown in Figure 2.

In step 5, we applied weighted cross-entropy as a loss function to train the model. The loss function is defined as follows:

(27) $$F_{l}=-[b\times \log(sigmoid(b*))\times \omega +(1-b)\times \log(1-siomoid(b*))]$$

where ω represents a weight parameter, which is equal to the ratio of negative samples to positive samples. This function is used to calculate the weighted cross-entropy between the true value of the label b and the target b* obtained by the model’s internal product algorithm. Figure 2 shows the GCN processing flow.

In step 6, the inner product algorithm based on the principle of matrix factorization (MF) was used to obtain the final score of each pair, and the reconstructed score matrix can be calculated as follows:

(28) $$S=sigmoid(M_{cm}M_{cm}^{⊤})$$

**Figure 3:**
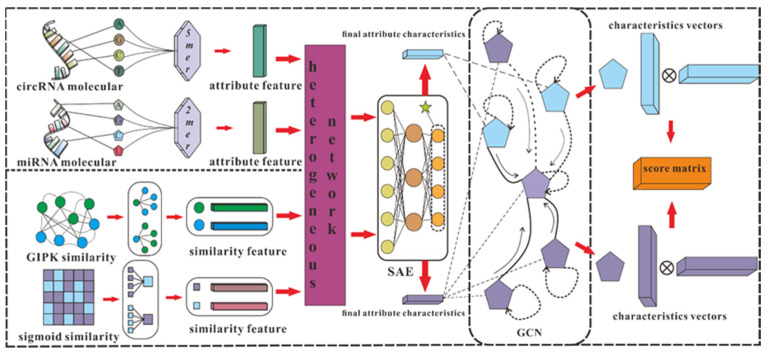
The detailed process of SGCNCMI.

The detailed process of SGCNCMI is shown in Figure 3.

In addition, our model directly uses the similarity of a marker to disease as an attribute feature when predicting the relationship between markers and underlying diseases due to the absence of molecular sequences. This makes our model more functional and robust as a predictor of both states.

## 3. Results

### 3.1. Evaluation Criteria

Cross-validation is an important evaluation method in the field of machine learning. This section describes the performance of the model as evaluated by five-fold cross-validation experiments. In the five-fold cross-validation, we first randomly divided the samples into five subsets; in each round of the cross-validation experiment, four subsets were used to train the model, and the last subsets were treated as the test set. Meanwhile, in order to ensure the comprehensiveness and fairness of the results and verify the stability and robustness of the model, we used frequently utilized metrics to fully validate our model, which are *Acc*. (Accuracy), Precision, and Recall. The calculation formula is defined as:

(29) $$Acc.=\frac{\left(TN+TP\right)}{\left(TN+TP+FN+FP\right)}$$

(30) $$\mathrm{Pr}ecision=\frac{TP}{\left(TP+FP\right)}$$

(31) $$Recall=\frac{TP}{\left(TP+FN\right)}$$

where TP (true positive) is the count of true samples predicted to have interacting circRNA–miRNA pairs; TN (true negative) is the number of true samples predicted to have non-interacting circRNA–miRNA pairs; FN (false negative) is the count of interacting circRNA–miRNA pairs that are predicted to have no interaction; and FP (false positives) refers to the number of non-interacting circRNA–miRNA pairs that are predicted to interact. In addition, AUC (the area under the ROC curve) and AUPR (the area under PR) were constructed to evaluate our model, and the mean value of five-fold cross-validation was used as the final score of the model.

### 3.2. Model Performance Evaluation

**Figure 4:**
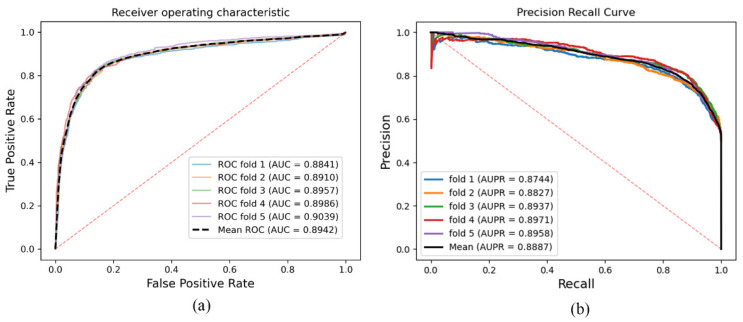
(**a**,**b**) are the ROC and PR curves generated by the SGCNCMI based on CMI-9905 dataset, respectively.

In this study, SGCNCMI was validated on the CMI-9905 dataset to evaluate the ability to predict potential circRNA–miRNA interactions. The results of the five-fold CV are recorded in Table 1. It can be seen in Table 1 that SGCNCMI achieved a mean AUC of 89.42% and a mean AUPR of 88.87%, where the AUCs of five-fold experiments were 88.41%, 89.10%, 89.57%, 89.86%, and 90.39%, and AUPR of each experiment was 87.44%, 88.27%, 89.37%, 89.71%, and 89.58%, respectively. The ROC curve and PR curve are plotted in Figure 4, which were generated by SGCNCMI using a five-fold CV.

**Figure 5:**
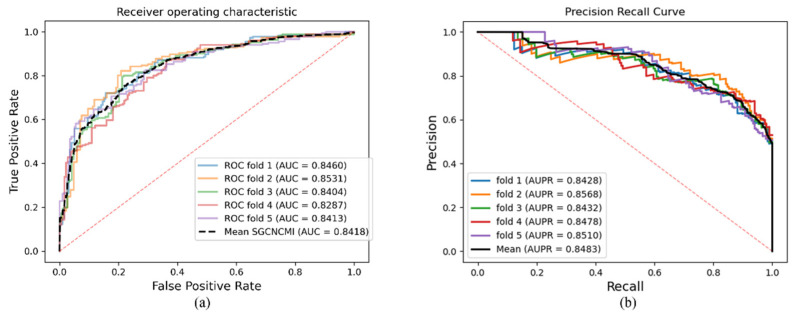
(**a**,**b**) are the ROC and PR curves generated by the SGCNCMI based on the circRNA–cancer dataset, respectively.

SGCNCMI’s biomarker–disease prediction results based on the circRNA–cancer dataset is presented in Table 2, and the AUC and ACPR curves are shown in Figure 5.

**Figure 6:**
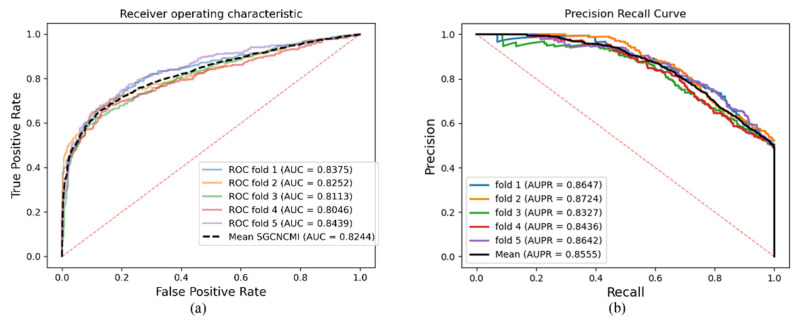
(**a**,**b**) are the ROC and PR curves generated by the SGCNCMI based on the circRNA–gene dataset, respectively.

SGCNCMI’s circRNA–gene prediction results based on the TransCirc dataset are presented in Table 3, and the AUC and ACPR curves are shown in Figure 6.

### 3.3. Discussion on the Effectiveness of GCN

The graph convolutional neural network (GCN) has been proven to be powerful for its ability to learn hidden features from an end-to-end model structure. In this work, we built a deep learning prediction model called SGCNCMI and introduced GCN into the model to aggregate the features of the relevant nodes in the network to mine hidden information for inferring circRNA–miRNA interactions.

**Figure 7:**
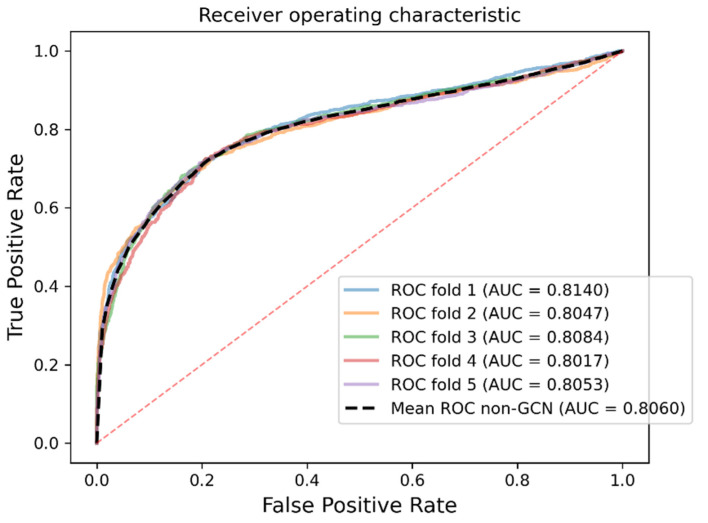
The ROC curves generated by SGCNCMI without GCN.

In order to express the effectiveness of GCN concretely, in this part, we evaluated the effectiveness of GCN concerning its ability to integrate the features of associated nodes. Specifically, we compared the feature extraction based on GCN with the case in which GCN is removed. To this aim, we removed the fourth step in SGCNCMI, and after the features were extracted by SAE, we directly carried out the sixth step to obtain the final prediction score of each circRNA–miRNA interaction pair. Using the inner product based on the matrix decomposition principle, we obtained the model results without GCN aggregation characteristics, which are shown in Table 4 and Figure 7.

Figure 7 shows that the model accuracy of GCN feature extraction has been greatly improved, which proves the effectiveness of GCN as a feature extraction link in the model. In addition, it is worth noting that our model still has good predictive performance without using GCN, which indicates that our model is scientific and efficient in extracting attribute node features. Meanwhile, the strategy for removing redundancy and extracting valid information from original features through SAE has been proven in previous studies [34].

### 3.4. Effect of the Number of GCN Layers

GCN is a graph neural network with a certain number of layers, and the network layer of the graph neural network projects the association graph into the spectral domain to aggregate the node information in the space. The number of convolutional layers plays a crucial role in aggregating node features and extracting potential information.

As described in this section, we established GCN models with different layers, namely, one layer, two layers, three layers, four layers, or five layers, for comparative observation and recording so as to explore the influence of different layers on feature aggregation.

**Figure 8:**
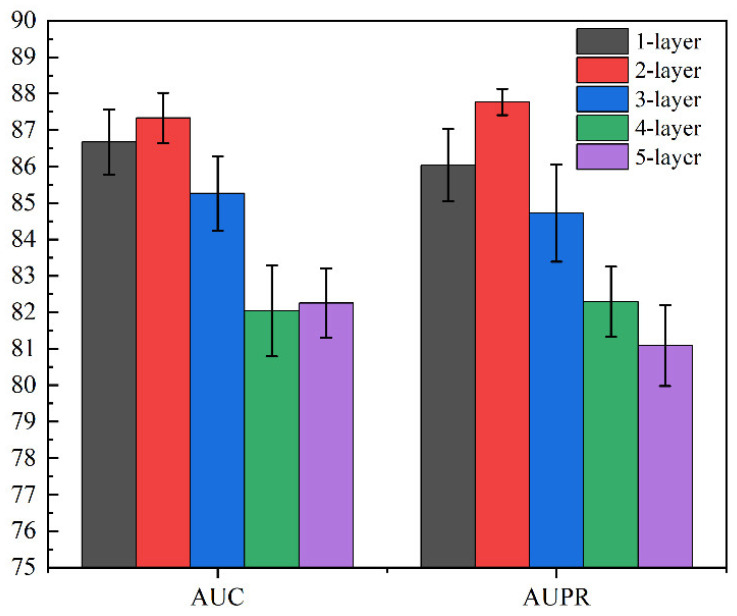
AUC and AUPR of SGCNCMI with different GCN layers.

Table 5 and Figure 8 show the AUC and AUPR of the model with different GCN layers. From the table, it is not difficult to find that GCN with one layer achieved great performance, which demonstrates the effectiveness of GCN. Next, GCN with two layers achieved the best performance, which indicates that the first two layers of GCN can effectively extract the hidden feature information of nodes. As the number of layers increased to three or more, the performance of the model began to deteriorate significantly, which may be due to the over-smoothing of GCN; at the same time, too many GCN layers may also lead to feature redundancy and “noise”.

Therefore, although GCN can effectively extract and aggregate node information, too few or too many layers will result in less-than-optimal results. Our experiments and records also provide a reference for the use of GCN by recording parameters of different layers.

### 3.5. Layer Attention Mechanism Analysis

Layer attention plays an important role in controlling and quantifying the contributions of different convolutional layers. Introducing a reasonable layer attention mechanism can maximize the contribution of each layer so as to obtain the best prediction effect.

**Figure 9:**
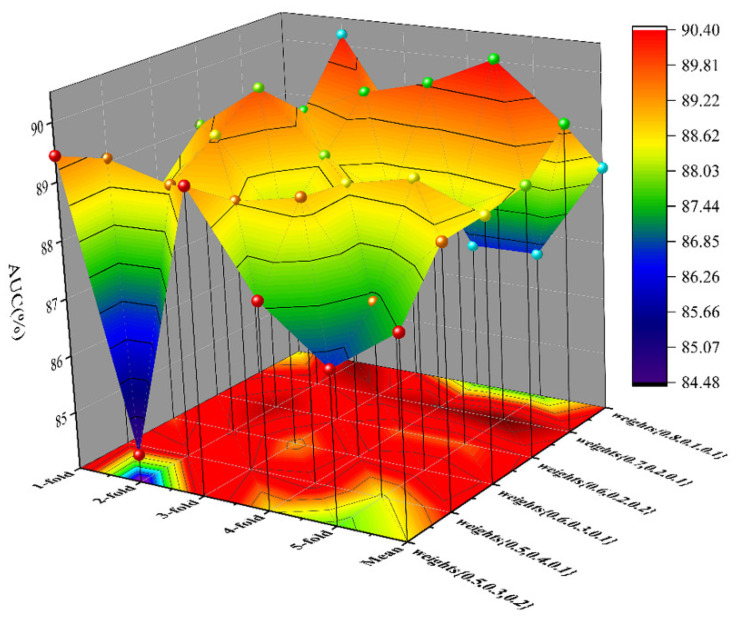
AUC of SGCNCMI with different parameters.

By building GCN models with different graph convolutional layers, we confirmed that each layer will have different effects on the model. In our model, the two-layer GCN model achieved the best results, which indicates that the first and second layers can effectively aggregate information. When the number of layers exceeded two, the performance of the model began to decline, and more layers often mean more redundant information, but this does not mean that these convolutional layers are not contributing. Therefore, assigning different attention weights to the convolutional layer is conducive to improving the contribution of the layers. Table 6 objectively lists the AUC of SGCNCMI with different parameters. To visually display the data, we projected the table into three-dimensional space, which is shown in Figure 9. Through the net pattern parameter, we assigned 0.7, 0.2, and 0.1 attention weights to three layers, respectively, and the model achieved the best performance.

### 3.6. Comparison of SGCNCMI with Other Related Models

Furthermore, in order to comprehensively prove the superiority of our model in the prediction of circRNA–miRNA interactions, we compared our model with existing models; specifically, we experimented with four models using a five-fold cross-validation method and the same dataset, and our model achieved the best effect. CMIVGSD [28] is the first calculation framework to predict circRNA–miRNA interactions, which obtains the score by using graph variational autoencoders and singular value decomposition. At present, there are few computational models about circRNA–miRNA interactions, so we also compared SGCNCMI with models in other highly relevant fields. The compared methods include DMFMDA [35], NTSHMDA [36], and AE_RF [37]. DMFMDA obtains a low-dimensional dense vector of microbes and diseases through a neural network and uses a neural network with an embedding layer for matrix factorization, and Bayesian Personalized Ranking is used to obtain the optimal model parameters. AE_RF integrates circRNA and disease similarities as features and extracts hidden biological patterns with a deep autoencoder, and the random forest classifier is used to predict the association. NTSHMDA obtains the heterogeneous network from a known microbe–disease association network by connecting the disease and microbe similarity network and uses random walk to predict human microbe–disease associations.

The specific comparison data are shown in Table 7. As shown in the table, our model results are 2% higher than those of the best model in the field of circRNA–miRNA interaction. Meanwhile, compared with models in other highly relevant fields, our model still has strong competitiveness. Without a doubt, SGCNCMI is one of the few powerful methods for predicting circRNA–miRNA interactions.

## 4. Case Studies

To verify the predictive ability of SGCNCMI under real conditions, we conducted a case study using 9905 circRNA–miRNA interaction pairs as a benchmark dataset. First, we used known circRNA–miRNA interaction pairs to build feature vectors and train the model. Next, the trained model was used to predict unknown interaction pairs. Finally, we ranked the final predicted scores from large to small. The top ten predicted scores are shown in Table 8. It can be seen in Table 8 that six of the top ten circRNA–miRNA interactions were confirmed in PubMed. The four unconfirmed pairs of interactions have not been confirmed by biological experiments, but the possibility of interaction between them is not ruled out.

## 5. Conclusions

Recently, accumulating experiments have shown that endogenous circRNAs can work as miRNA sponges, which means that circRNAs bind to miRNAs and repress their functions [38]. Predicting circRNA–miRNA interactions reveal a new mechanism for regulating miRNA activity, which will benefit the diagnosis and treatment of diseases. Predicting circRNA–miRNA interactions by the computational method can not only reduce experimental risk and cost but also provide specific ideas for biological experiments. In this work, we developed a computational model named SGCNCMI to predict potential associations based on known associations. In the model, we construct molecular signatures from a variety of angles and use SAE to extract and fuse the features. Then, based on the known association diagram, the association information of surrounding nodes is fully aggregated by a graph convolutional neural network. Finally, the predicted score is obtained through the inner product decoder. We used a variety of evaluation indicators to evaluate the predictive performance of the model, which proved that our model can effectively predict potential circRNA–miRNA interactions. At the same time, our model achieved the best results in the field of predicting circRNA–miRNA interactions, and the performance was better than the only known model at present. Our model shows promising results in predicting both circRNA–cancer and circRNA–gene associations, meaning that our model can be used not only at the molecular level but also for the diagnosis of clinical diseases and the discovery of potentially associated genes, demonstrating the power of our model.

Limited by the number and availability of datasets, the application of computational methods in the field of circRNA–miRNA interaction prediction is in its infancy, and our model is the second known calculation method. In this work, we not only carried out experiments on the data of previously published methods but also improved and added some new reliable data. In the future, we will continue to collect more comprehensive and reliable data and propose new effective computational methods with higher performance. With circRNA becoming a new hotspot in RNA research, new methods will be constantly proposed, and our model will certainly provide a reference for more reliable methods in the future.

## Figures and Tables

**Table 1:** Five-fold cross-validation results based on CMI-9905 performed by SGCNCMI.

SGCNCMI	One-Fold	Two-Fold	Three-Fold	Four-Fold	Five-Fold	Mean
AUC	0.8841	0.8910	0.8957	0.8986	0.9039	0.8942
AUPR	0.8744	0.8827	0.8937	0.8971	0.8958	0.8887

**Table 2:** Five-fold cross-validation results of circRNA–cancer performed by SGCNCMI.

SGCNCMI	One-Fold	Two-Fold	Three-Fold	Four-Fold	Five-Fold	Mean
AUC	0.8460	0.8531	0.8404	0.8287	0.8413	0.8418
AUPR	0.8428	0.8568	0.8432	0.8478	0.8510	0.8483

**Table 3:** Five-fold cross-validation results of circRNA–gene performed by SGCNCMI.

SGCNCMI	One-Fold	Two-Fold	Three-Fold	Four-Fold	Five-Fold	Mean
AUC	0.8375	0.8252	0.8113	0.8046	0.8439	0.8244
AUPR	0.8647	0.8724	0.8327	0.8436	0.8642	0.8555

**Table 4:** Five-fold cross-validation results obtained by SGCNCMI without GCN.

Non-GCN	One-Fold	Two-Fold	Three-Fold	Four-Fold	Five-Fold	Mean
AUC	0.8140	0.8047	0.8084	0.8017	0.8053	0.8060
AUPR	0.8454	0.8441	0.8408	0.8258	0.8412	0.8393

**Table 5:** AUC and AUPR of SGCNCMI with different GCN layers.

Layers	One Layer	Two Layers	Three Layers	Four Layers	Five Layers
AUC	0.8667	0.8733	0.8526	0.8204	0.8225
AUPR	0.8604	0.8777	0.8472	0.8229	0.8109

**Table 6:** AUC of SGCNCMI under different parameters.

	AUC	One-Fold	Two-Fold	Three-Fold	Four-Fold	Five-Fold	Mean
Weights							
{0.5,0.3,0.2}		89.44	84.48	89.25	87.54	86.62	87.43
{0.5,0.4,0.1}		89.07	88.77	88.65	88.88	87.32	88.49
{0.6,0.3,0.1}		88.19	89.29	87.88	88.77	89.01	88.56
{0.6,0.2,0.2}		89.02	89.81	88.76	88.15	88.13	88.71
{0.7,0.2,0.1}		88.41	89.10	89.57	89.86	90.39	89.42
{0.8,0.1,0.1}		87.28	90.20	88.49	86.61	86.63	88.34

**Table 7:** Results of comparison with highly relevant models.

Methods	AE_RF	DMFMDA	NTSHMDA	CMIVGSD	SGCNCMI
AUC	0.7662	0.7922	0.8526	0.8804	0.9015
AUPR	0.8239	0.8230	0.8772	0.8629	0.9011

**Table 8:** The top ten prediction results in SGCNCMI based on the dataset.

Num	circRNA	miRNA	Evidence
1	hsa_circ_0003998	hsa-miR-326	PMID:30764896
2	hsa_circ_0000523	hsa-miR-31	PMID:30403259
3	hsa_circ_0044553	hsa-miR-4726-5p	Unconfirmed
4	hsa_circ_0000554	hsa-miR-339-5p	PMID:27465405
5	hsa_circ_0089776	hsa-miR-6752-5p	Unconfirmed
6	hsa_circ_0061537	hsa-miR-3913-3p	Unconfirmed
7	hsa_circ_0010596	hsa-miR-660-3p	PMID:32584784
8	hsa_circ_0000799	hsa-miR-31-5p	PMID:30103209
9	hsa_circ_0068761	hsa-miR-4487	PMID:33534927
10	hsa_circ_0079155	hsa-miR-6802-3p	Unconfirmed

## Data Availability

The datasets described in this paper can be found in Circbank http://www.circbank.cn/ (accessed on 11 July 2022) and CircR2Cancer http://www.biobdlab.cn:8000/ (accessed on 11 July 2022). The data and source code can be found at https://github.com/1axin/SGCNCMI (accessed on 11 July 2022).
